# Hyperparameter optimisation and validation of registration algorithms for measuring regional ventricular deformation using retrospective gated computed tomography images

**DOI:** 10.1038/s41598-021-84935-x

**Published:** 2021-03-11

**Authors:** Orod Razeghi, Mattias Heinrich, Thomas E. Fastl, Cesare Corrado, Rashed Karim, Adelaide De Vecchi, Tom Banks, Patrick Donnelly, Jonathan M. Behar, Justin Gould, Ronak Rajani, Christopher A. Rinaldi, Steven Niederer

**Affiliations:** 1 School of Biomedical Engineering and Imaging Sciences, King’s College London, London, UK; 2 Insitute of Medical Informatics, University of Lübeck, Lübeck, Germany; 3 Department of Cardiology, Guy’s and St Thomas’ NHS Foundation Trust, London, UK; 4 South Eastern Health and Social Care Trust, Dundonald, Belfast, UK; 5 Department of Electrophysiology, Barts Heart Centre, London, UK

**Keywords:** Cardiology, Computed tomography, Image processing, Software

## Abstract

Recent dose reduction techniques have made retrospective computed tomography (CT) scans more applicable and extracting myocardial function from cardiac computed tomography (CCT) images feasible. However, hyperparameters of generic image intensity-based registration techniques, which are used for tracking motion, have not been systematically optimised for this modality. There is limited work on their validation for measuring regional strains from retrospective gated CCT images and open-source software for motion analysis is not widely available. We calculated strain using our open-source platform by applying an image registration warping field to a triangulated mesh of the left ventricular endocardium. We optimised hyperparameters of two registration methods to track the wall motion. Both methods required a single semi-automated segmentation of the left ventricle cavity at end-diastolic phase. The motion was characterised by the circumferential and longitudinal strains, as well as local area change throughout the cardiac cycle from a dataset of 24 patients. The derived motion was validated against manually annotated anatomical landmarks and the calculation of strains were verified using idealised problems. Optimising hyperparameters of registration methods allowed tracking of anatomical measurements with a mean error of 6.63% across frames, landmarks, and patients, comparable to an intra-observer error of 7.98%. Both registration methods differentiated between normal and dyssynchronous contraction patterns based on circumferential strain ($p_1=0.0065$, $p_2=0.0011$). To test whether a typical 10 temporal frames sampling of retrospective gated CCT datasets affects measuring cardiac mechanics, we compared motion tracking results from 10 and 20 frames datasets and found a maximum error of $8.51\pm 0.8\%$. Our findings show that intensity-based registration techniques with optimal hyperparameters are able to accurately measure regional strains from CCT in a very short amount of time. Furthermore, sufficient sensitivity can be achieved to identify heart failure patients and left ventricle mechanics can be quantified with 10 reconstructed temporal frames. Our open-source platform will support increased use of CCT for quantifying cardiac mechanics.

## Introduction

Global cardiac mechanics, routinely measured using ejection fraction, plays a significant role in the diagnosis and stratification of cardiology patients. However, the heart pathology does not always manifest itself as a change in global function. Owing to to advances in image reconstruction techniques and progressive improvements in both spatial but specifically the temporal resolution of dual-source CT acquisition sequences, it is now feasible to measure myocardium motion regionally throughout the cardiac cycle and augment the established global measurements^1^.

Historically, measuring regional cardiac mechanics has focused on characterising motion using two and three dimensional echocardiography^2^ and cardiac magnetic resonance (CMR) imaging^1,3–5^. In these modalities, multiple images of the heart throughout the cardiac cycle are generated and features (speckle, tags or anatomy) are tracked between frames. However, the current growing number of patients, who are contraindicated from magnetic resonance imaging (MRI) due to their implanted devices, is leading to an enhanced demand for motion tracking from cardiac computed tomography (CCT) images. Simultaneously, recent reductions in the radiation dose techniques such as dose modulation, denoising algorithms, and iterative reconstruction have lowered the risk of using computed tomography (CT), increasing the potential applications of this modality^6,7^.

In order to establish the correlation between regional function estimates from CMR and CCT, Pourmorteza et al.^8,9^ tracked the motion of the left ventricular endocardium to measure regional deformation. They calculated local area change and labelled it “SQUEEZ”. Their method relied on a non-rigid point registration algorithm termed coherent point drift (CPD) for warping surfaces representing end-diastole to end-systole^10^. They did not attempt to measure circumferential or longitudinal strains, nor was their tracking validated against manual annotations. Another example of a point set registration framework used to measure local area change is the loop subdivision surface method^11^, which was originally introduced for cardiac modelling and segmentation in the context of 3D echocardiography. Vigneault et al.^12^ adapted this approach, used the simultaneous subdivision surface registration (SiSSR) method to measure SQUEEZ in 13 canine hearts, and compared the results against the CPD technique. However, their method was not applied to clinical datasets and their selected registration method requires binary segmentation of the blood pool and mesh generation from every phase of the cardiac cycle. They used thresholding and morphological techniques to extract these meshes, which can be potentially a subjective and time consuming task for cardiologists, limiting the clinical translation of the technique.

Thus far, feature tracking methods from echocardiography^11^ and point sets registration techniques^10^ have been applied to CCT. However, limited systematic optimisation has been performed on the significant investment of algorithms used for feature tracking in CMR images, nor on the CT registration techniques developed for tracking motion in other organs. CCT can offer isotropic voxels and higher image resolution compared to echocardiography and CMR. The resulting image stacks have a typical size of 512x512x250 voxels over 10 to 20 frames, which makes them on average an order of magnitude larger than their CMR counterparts. This can potentially increase the computational cost for most image registration techniques and therefore fine tuning for the specific clinical application may become important. For example, the recent estimation of CCT motion displacements from 10 patients using the deformable image registration approach in Gupta et al.^13^ took approximately 35 minutes per subject over the entire cardiac cycle, which is not ideal for incorporation in a clinical workflow.

Apart from finding the optimal registration algorithm’s hyperparameters, it is important to test its performance on validation data. Lamash et al.^14^ work is amongst few studies on validation of strains computed from CCT. They validated their algorithm on 27 patients, of which only 12 were diagnosed as abnormal. Their results were evaluated against values from 2D speckle tracking analysis and visual scores obtained by an expert. However, there was no direct validation of the performed registration on the CCT datasets. Accurate measures of regional mechanics are particularly valuable in diagnosing and treating patients with dyssynchronous heart failure, who are receiving cardiac resynchronisation therapy (CRT). During this procedure, pacing leads are placed on the left and right hand side of the heart to synchronise electrical, and hence mechanical, cardiac function. Lead location is therefore recognised as an important factor in patient outcome. Regional mechanical function estimated entirely from endocardial surface has been previously used for identification of the optimal lead location, which represented an early use case of CCT motion tracking for guiding implants in patients receiving an upgrade to CRT^15^.

In this paper, we briefly introduce our open-source clinician-focused platform developed to process CCT datasets. We optimise the temporal sparse free-form deformations (TSFFD) method^16^ and the dense displacement sampling (DEEDS) registration tool^17^. TSFFD is selected because it is based on the widely used non-rigid registration approach of Rueckert et al.^18,19^ and has been previously used to track motion in CMR. DEEDS, on the other hand, has been comprehensively evaluated on 100 abdominal CT scans for inter-patient registration and has achieved the highest accuracy for 13 anatomical structures in comparison to several state-of-the-art approaches^17^. We extend these registration methods with a point set transformation algorithm to deform triangulated meshes. Furthermore in this paper, we derive area strains using the method described in Pourmorteza et al.^8^ as well as circumferential and longitudinal strains using large strain theory across clinical datasets of 24 patients. We validate and compare the two algorithms by testing their ability to differentiate dyssynchronous heart failure patients against healthy controls using anatomically annotated images and evaluate the impact of frame rate on strain calculations. Our optimised pipeline is able to register an entire patient’s CCT dataset in approximately five minutes on a CPU, which is compatible for use in an interactive clinical workflow.

## Methods

Characterising cardiac wall motion requires a sequence of image processing steps, which we describe in this section. We first introduce our designed workflow, then review the clinical datasets used in our experiments, and finally conclude with the description of a cost function, which was implemented to validate the accuracy of registration techniques.

### Workflow

**Figure 1:**
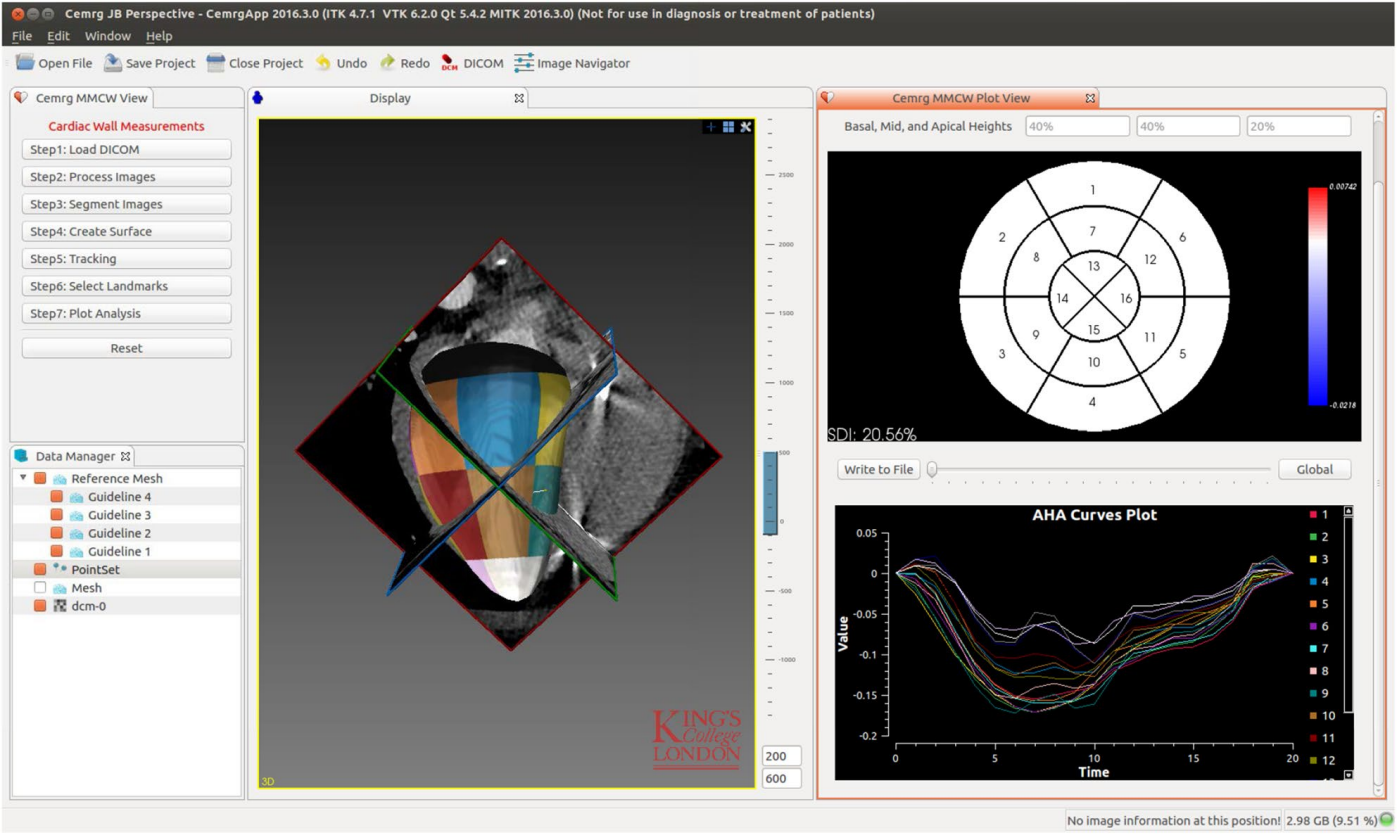
Our platform is based on MITK features and provides a straightforward approach for analysing wall motion from CCT datasets. The window in the top right corner displays a 16-segment bullseye plot for visualisation of strain at every phase in the cardiac cycle. The window in the bottom right corner plots individual strain curves from the endocardial segments. The larger window in the left is an interactive renderer for visualising 3D images and surface meshes.

A workflow was designed for and tested by cardiologists to compute left ventricular endocardial strains. The workflow was developed as a platform based on utilities of the Medical Imaging Interaction Toolkit (MITK) framework (http://www.mitk.org). Linux, macOS, and Microsoft Windows binary distributions of our platform, as well as its source code, is publicly available from our website (http://www.cemrgapp.com). Figure 1 illustrates the graphical user interface (GUI) of our developed platform with all the processing steps summarised by seven simple push buttons in the left hand panel. The following describes the components of this workflow.

#### Image processing and mesh generation

Initially, CCT datasets were converted from their original DICOM (http://medical.nema.org) format into a series of NIfTI (http://nifti.nimh.nih.gov) images. Dimension of images varied between datasets but the majority were 512 x 512 voxels with a 0.32–0.48 mm isotropic in plane resolution. The 3D stacks had 121–365 slices with a through plane thickness of 0.8–2 mm. The Hounsfield unit spanned from $\text {-}1024-3071$. Each dataset consisted of 10 images equally spaced throughout the cardiac cycle with a temporal resolution spanning 72–120 ms depending on the patient’s heart rate. Ventricular cavities were cropped using a user defined bounding box to reduce image size and improve processing times. The reference image was selected from the first frame of the CCT, representing the heart at the end-diastolic phase and the peak of the R-wave in the electrocardiogram (ECG). Next, the blood pool of the LV including the papillary muscles was segmented from the reference image. Segmentation was performed using a grey value based region growing tool. The grey values were determined from all point positions plus/minus a margin of 30 Hounsfield units. The 2D region growing segmentation was applied in 5–10 of the long axis slices. These slices were interpolated to label the LV cavity in 3D with the option for manual correction. After achieving a full segmentation, a marching cubes process generated a smooth endocardial surface from the segmentation. Finally, six anatomical landmarks were selected on the reference image: one on the apex, three on the surface of the mitral valve and two on the septum, delineated by the attachment of the right ventricle. These landmarks defined a coordinate system that could be used to label sections of the reference mesh surface with one of the AHA segments^20^. These segments enabled local interpretation of the analysis.

#### Endocardium motion estimation

Tracking endocardial motion can essentially be defined as the non-rigid registration of cardiac image sequences. We optimised the following two registration algorithms to track motion of the LV endocardium from the series of images processed previously. Their full set of hyperparameters can be found in the supplements.

#### Temporal sparse free-form deformation

In free-form deformation (FFD) registration^18^, a non-rigid deformation $\pmb {h} = [X\ Y\ Z]^T$ is represented using a B-spline model in which the deformation is parametrised using a set of control points $\pmb {\Phi } = [U\ V\ W]^T$. To be able to deal with large global deformations and to improve the robustness, the classic FFD registration normally uses a multi-level representation^19^. We used the sparse free-form deformation (SFFD) technique^16^ to extend the classic FFD approach and recover smoother displacement fields. The assumption is that a typical FFD with dense displacement can be sparse in its parametric representation. To model the cyclic deformation in an image sequence with $N_{t}$ temporal frames, a set of 4D control points were constructed, which could further extend the sparsity constraint to the temporal domain for a temporal sparse free-form deformation framework. In our workflow, we optimised and validated this technique by using a four-level representation and sum of squared differences as the similarity measure. The registration energy function was minimised using a gradient descent approach^21,22^.

#### Dense displacement sampling registration

As an alternative tracking technique, we adapted the dense displacement sampling registration method introduced in Heinrich et al.^17^ In this technique, a graph is defined in which the nodes $p\in \mathcal{P}$ correspond to control points in a uniform B-spline grid. For each node in the graph, there is a set of labels $f_{p}$, which correspond to a set of discrete 3D displacements: $f_{p} = \mathbf{u}_{p} = \{u_{p}\ v_{p}\ w_{p}\}$. This technique uses a contrast and modality-invariant similarity metric based on binarised self-similarity context (SSC) descriptors^23^. We relied on a dense displacement search and performed five iterations on a single high-resolution image with different grid-spacings. These control-point spacings were by default defined as 8 x 7 x 6 x 5 x 4 voxels for each level. The neighbourhood relations of the control point grid were approximated using a minimum-spanning-tree to find a smooth displacement field.

#### Reference mesh deformation

The displacement fields, generated by the registration algorithms, were required to deform the reference mesh and produce a series of meshes corresponding to each phase of the cardiac cycle. To augment the original registration frameworks with a point set transformation algorithm, we utilised a linear function to interpolate the computed control point displacement fields: $\Pi (M,\mathbf{u}_{p})$. The interpolated fields were then used to deform the reference mesh by:

1 $$\begin{aligned} M^{Deformed} = {\left\{ \begin{array}{ll} m^{Reference}_x + \Pi (m^{Reference}_x, u_{p}) \\ m^{Reference}_y + \Pi (m^{Reference}_y, v_{p}),\\ m^{Reference}_z + \Pi (m^{Reference}_z, w_{p}) \end{array}\right. } \end{aligned}$$

where $M = \{m_{x}, m_{y}, m_{z}\}$ is a point in the set of all mesh point coordinates.

#### Calculation of strain on endocardium

Cardiac deformation is the transformation of the endocardial reference mesh into a configuration representing each phase of the cardiac cycle. To calculate circumferential and longitudinal strains from each of these configurations, an element coordinate system was defined by normalised base vectors:

2 $$\begin{aligned} \mathbf{e}_{\mathrm{r}}&= \frac{\mathbf{e}_{21} \times \mathbf{e}_{31}}{\Vert \mathbf{e}_{21} \times \mathbf{e}_{31}\Vert }, \end{aligned}$$

3 $$\begin{aligned} \mathbf{e}_{\mathrm{z}}&= \frac{\mathbf{v}_{\mathrm{ab}}-(\mathbf{v}_{\mathrm{ab}} \cdot \mathbf{e}_{\mathrm{r}})\mathbf{e}_{\mathrm{r}}}{\Vert \mathbf{v}_{\mathrm{ab}}-(\mathbf{v}_{\mathrm{ab}} \cdot \mathbf{e}_{\mathrm{r}})\mathbf{e}_{\mathrm{r}}\Vert }, \end{aligned}$$

4 $$\begin{aligned} \mathbf{e}_{{\theta }}&= \frac{\mathbf{e}_{\mathrm{z}} \times \mathbf{e}_{\mathrm{r}}}{\Vert \mathbf{e}_{\mathrm{z}} \times \mathbf{e}_{\mathrm{r}}\Vert }, \end{aligned}$$

where $\mathbf{e}_{21}$ and $\mathbf{e}_{31}$ are the mesh element edge vectors between subscript vertices (note the counter-clockwise triangular element numbering) and $\mathbf{v}_{\mathrm{ab}} = \mathbf{X}_{\mathrm{b}}-\mathbf{X}_{\mathrm{a}}$ represents the left ventricular long axis defined by the apical and basal points $\mathbf{X}_{\mathrm{a}}$ and $\mathbf{X}_{\mathrm{b}}$, respectively. The clinically used cylindrical element coordinate system with circumferential and longitudinal components was defined by rotating the Green-Lagrange strain tensor $\mathbf{E}$ from the global Cartesian coordinate system. The cylindrical element coordinate system remained the same for every time frame in the cardiac cycle and the rotation was utilised by the transformation matrix $\mathbf{Q}$ evaluated as:

5 $$\begin{aligned} \mathbf{E}_{\mathrm{e}} = \mathbf{Q}{} \mathbf{E}{} \mathbf{Q}^{\mathrm{T}}, \quad \mathrm{with} \quad \mathbf{Q} = \begin{bmatrix} \mathbf{e}_{\mathrm{r}} \cdot \mathbf{e}_{\mathrm{1}} &{} \mathbf{e}_{\mathrm{r}} \cdot \mathbf{e}_{\mathrm{2}} &{} \mathbf{e}_{\mathrm{r}} \cdot \mathbf{e}_{\mathrm{3}}\\ \mathbf{e}_{\mathrm{\theta }} \cdot \mathbf{e}_{\mathrm{1}} &{} \mathbf{e}_{\mathrm{\theta }} \cdot \mathbf{e}_{\mathrm{2}} &{} \mathbf{e}_{\mathrm{\theta }} \cdot \mathbf{e}_{\mathrm{3}}\\ \mathbf{e}_{\mathrm{z}} \cdot \mathbf{e}_{\mathrm{1}} &{} \mathbf{e}_{\mathrm{z}} \cdot \mathbf{e}_{\mathrm{2}} &{} \mathbf{e}_{\mathrm{z}} \cdot \mathbf{e}_{\mathrm{3}} \end{bmatrix}, \end{aligned}$$

where $\mathbf{e}_{i}$, for $i = 1,2,3$, represents the unit direction vector in the Cartesian coordinate system. After the rotation, the radial strain components vanished, i.e. $E_{\mathrm{r}i} = E_{i\mathrm{r}} = 0$, due to the definition of the problem. The other elements in the main diagonal of the strain tensor, i.e. $E_{\theta \theta }$ and $E_{\mathrm{zz}}$, provided us with the circumferential and longitudinal strains for a given element. Regional strains were the mean of the individual elements strains in each AHA segment. Full details on calculating deformation gradient tensor **F** and the Green-Lagrangian strain tensor **E** can be found in the supplements. We also verified the mathematical description of these tensors using idealised problems in the supplements.

### Clinical data

The clinical data in our experiments were previously used in a study by Banks et al.^24^ Eighteen of the CCT scans were performed on patients undergoing CRT. For verification of the motion tracking algorithms, we also obtained a further set of six healthy control sets, which were diagnostically scanned but returned no indication of cardiac disease. Scans were performed using a Philips Brilliance iCT 256-slice MDCT scanner (Philips Healthcare, Amsterdam, Netherlands). Intravenous metoprolol was used to achieve a mean heart rate of $64\pm 7$ beats/min. The mean radiation dose-area product was $1194\pm 419$ mGy cm^2^. A total of 100 ml of intravenous contrast agent (Omnipaque, GE Healthcare, Princeton, NJ, USA) was injected via a power injector into the antecubital vein. Helical scanning was performed with a single breath-hold technique after a 10–12 s delay. The scanning parameters included: a heart rate dependent pitch of 0.2–0.45, a gantry rotation time of 270ms, a tube voltage of 100 or 120 kVp depending on the patient’s body mass index, and a tube current of 125–300 mA depending upon the thoracic circumference. Retrospectively ECG-gated image reconstruction was used to generate 10 images per cardiac cycle.

Data were collected in accordance with relevant guidelines and regulations as part of two clinical trials, which were approved by the West Midlands Coventry & Warwick REC (14/WM/1069) and the London-Harrow (18/LO/0752) ethics committees. All the patients gave written informed consent and the scans were analysed anonymously. Healthy control data was obtained through the South Eastern Health and Social Care trust. The data was fully anonymised and used with consent from the trusts data governance.

The quality of measuring motion from CCT scans is predominately dependent on the efficacy of our selected registration algorithms. Hence, changes in their hyperparameters values have a considerable effect on the outcome of the strain computations, as neither of them were developed for CCT. To create training and validation sets for optimising their hyperparameters, all CRT cases had to be annotated with anatomical landmarks by a trained clinician. We randomly separated the datasets in 10 training and 5 validation sets, resulting in 100 and 50 annotated temporal frames, respectively. Out of 18 datasets, 3 could not be landmarked confidently due to the low image quality.

### Tracking error definition

**Figure 2:**
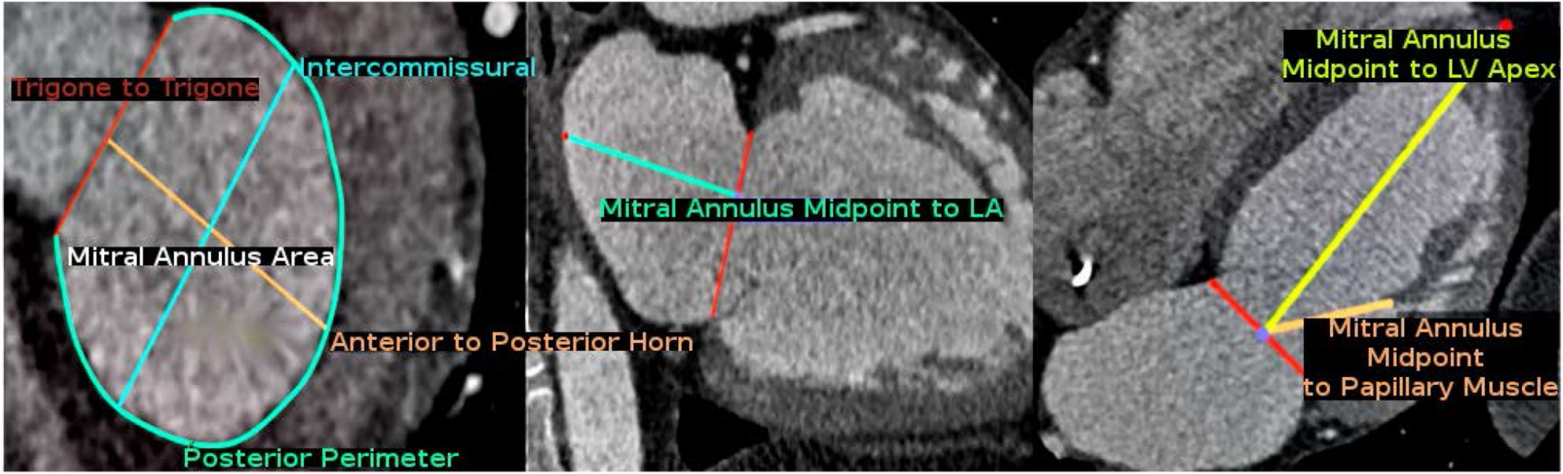
Illustration of the landmarks taken in the short axis, 2-chamber, and 4-chamber views of a CCT scan. Measurements were calculated from the analysis of the followings: (1) trigone to trigone diameter, (2) posterior perimeter, (3) mitral annulus area, (4) intercommissural diameter, (5) anterior to posterior horn diameter, (6) mitral annulus midpoint to head of the anterolateral papillary muscle (PM), (7) mitral annulus midpoint to head of the posteromedial papillary muscle (PM), (8) mitral annulus midpoint to endocardial border of the LV apex, (9) mitral annulus midpoint to epicardial border of the LV apex, and (10) mitral annulus midpoint to posterior wall of the left atrium (LA).

To define a tracking error, the clinician initially annotated the dataset with anatomical landmarks manually^24^ using the OsiriX software package^25^. All of the landmarks were performed at phase 0% of the cardiac cycle and then repeated through all of the remaining phases to give a total number of 100 measurements for one scan. These measurements are illustrated and described in Fig. 2.

Subsequently, the same measurements were repeated within our workflow but only on the phase 0% of the CCT sets and the remaining nine phases were automatically calculated by the registration algorithm after tracking the cardiac motion. Each registration algorithm was optimised based on the anatomical measurements available in the training sets. We defined tracking error as the relative difference between the observed *y* and the estimated $h_\Theta (x)$ anatomical measurements. Since there are 10 sets of anatomical measurements and each measurement can be examined in all 10 possible temporal frames, we set the cost function for optimising our selected registration algorithms to be the mean across all the time frames:

6 $$\begin{aligned} J(\Theta ) = \frac{1}{mt}\sum _{i=1}^{m}\sum _{j=1}^{t}(h_{\Theta }(x) - y)^2, \end{aligned}$$

where $\Theta$ is the set of parameters for a selected registration algorithm, *t* is the number of temporal frames per scan, and *m* is the training sets.

## Results

In this section, we initially look into the registration optimisation and its evaluation on the training sets. We further verify our tracking error on the validation sets and then explore the results of calculated strains for CRT patients and healthy controls. We finish by examining the effect of number of reconstructed temporal frames on the calculated results.

### Registration optimisation and evaluation

**Figure 3:**
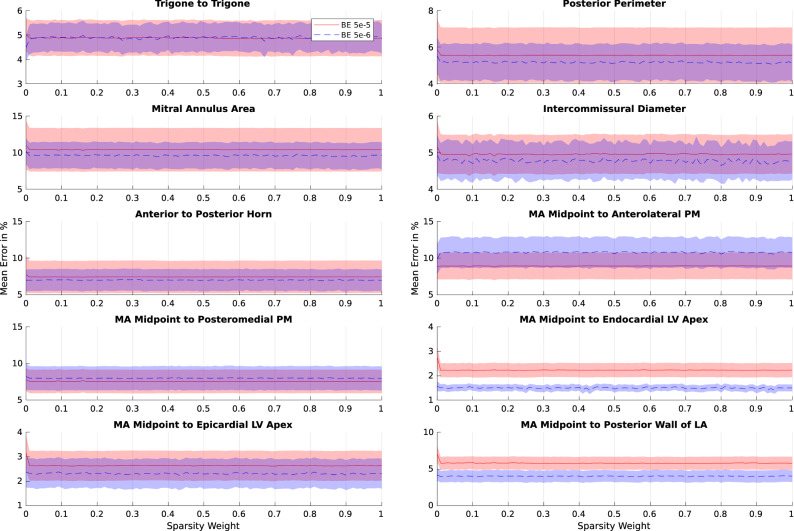
$\mathbf {X}$ axis is the examined range for the sparsity weight optimisation, whereas $\mathbf {Y}$ axis displays the average error in percentage on the training sets. The two curves in the plot correspond to the two values for the bending energy. Standard error is illustrated as a shaded region. Sparsity weight does not show a significant effect on the accuracy.

TSFFD method has two important hyperparameters: bending energy and sparsity weight. Bending energy regularisation $\Theta _{0}$ is applied between neighbouring control points within each FFD level to encourage a grouped sparse solution. The sparsity weight $\Theta _{1}$ is introduced into the deformation to enforce coupled multi-level sparsity. We initially took an exhaustive grid search strategy with no preconceived bias and examined 121 different permutations of these hyperparameters spanning from 0.0 to 1.0 with a resolution of 0.1. However, manual variation of the bending energy $\Theta _{0}$ revealed that a balance between limited motion and tracking of noise could be established within the range of $10^{-6}$ and $10^{-5}$. To evaluate which of these two values was optimal for the sparsity weight $\Theta _{1}$, we performed 202 further evaluations with a 0.01 resolution. Sparsity weight did not show any significant effect on the accuracy of measurements. The refined grid search can be seen in Fig. 3 and details of the initial exhaustive search with the full span of values can be found in the supplements.

DEEDS deformable registration is formulated as Markov random fields and the neighbourhood relations of the control point grid are approximated using a minimum-spanning-tree to find a smooth displacement field given an additional registration hyperparameter $\Theta$. The value can be increased to obtain smoother transforms and decreased to make the registration more aggressive. Similar to the previous algorithm, we designed a full grid search approach to find the optimal value with 21 permutations. This hyperparameter had a moderate influence on the accuracy of measurements. Details of the grid search can be found in the supplements.

### Tracking error in validation sets

**Figure 4:**
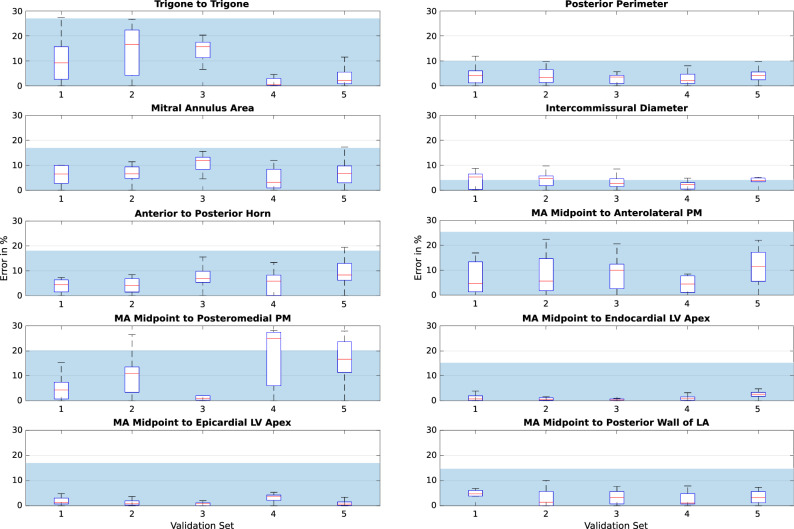
TSFFD tracking error, where $\mathbf {X}$ axis is the validation set. $\mathbf {Y}$ axis displays the error calculated in percentage. Each box plot represents the error in percentage from all the 500 measurements throughout the cardiac cycle. Mean intra-observer error across all sets is illustrated as a shaded region.

**Figure 5:**
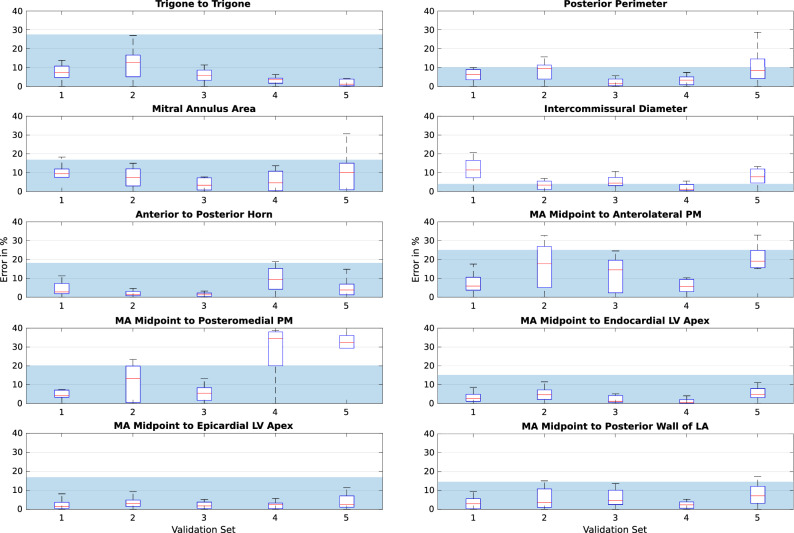
DEEDS tracking error, where $\mathbf {X}$ axis is the validation set. $\mathbf {Y}$ axis displays the error calculated in percentage. Each box plot represents the error in percentage from all the 500 measurements throughout the cardiac cycle. Mean intra-observer error across all sets is illustrated as a shaded region.

We evaluated the accuracy of both registration algorithms across 50 temporal frames and 500 measurements after optimising the hyperparameters based on our findings in the previous section. Bending energy and sparsity weight were set as $5\mathrm {e}{-6}$ and 0.43, respectively for the TSFFD algorithm. The registration hyperparameter of DEEDS was fixed as 0.5. Figure 4 illustrates the error of the TSFFD algorithm in tracking the anatomical measurements across frames for all the validation sets. Figure 5 shows similar results for the DEEDS algorithm. The manual measurements were carried out twice on the first frame of the CCT sets using two separate platforms. Computation of intra-observer variability therefore provided us with a measure of reproducibility in selecting these anatomical landmarks manually. We calculated the intra-observer error by defining the manual measurements on the OsiriX platform as the ground truth. The shaded regions in the two validation figures are the average intra-observer error across all validation sets. Optimisation of hyperparameters allowed tracking of anatomical measurements with mean errors of 5.99% and 7.27% across frames, landmarks, and patients for TSFFD and DEEDS, respectively. These results were comparable to an intra-observer error of 7.98%. We further examined the intra-observer variability by utilising a two-sample *t*-test to compare the anatomical landmarks placed on the first frames and find the least reproducible measurements. Amongst the calculated measurements, *Trigone to Trigone* and *Mitral Annulus Midpoint to head of the Anterolateral Papillary Muscle* were significantly different on each platform with 0.0184 and 0.0179 *p*-values and 95% confidence intervals of [0.44, 4.44] and [0.76, 7.5], respectively.

We performed further correlation tests to compare manual measurements from the analysis of anatomical landmarks to automatic measurements calculated from tracking of the same landmarks using the two registration algorithms. Moderate and strong correlations for majority of these measurements were confirmed by calculating the Pearson Correlation Coefficient and Coefficient of Determination values in the validation sets. Table 1 lists the individual results.

**Table 1:** Manual measurements from analysis of anatomical landmarks are compared to measurements calculated from automatic tracking of the same landmarks using our two registration algorithms.

Registration algorithm	TSFFD		DEEDS	
Correlation method	*r*	$R^2$	*r*	$R^2$
Trigone to trigone	0.61	0.38	0.67	0.45
Posterior perimeter	0.77	0.60	0.66	0.44
Mitral annulus area	0.83	0.69	0.71	0.51
Intercommissural diameter	0.77	0.60	0.64	0.41
Anterior to posterior horn	0.74	0.55	0.66	0.44
MA midpoint to anterolateral PM	0.66	0.44	0.59	0.35
MA midpoint to posteromedial PM	0.85	0.73	0.61	0.38
MA midpoint to endocardial LV apex	0.86	0.74	0.78	0.61
MA Midpoint to epicardial LV apex	0.76	0.59	0.68	0.46
MA Midpoint to posterior wall of LA	0.81	0.66	0.66	0.44

The results show a moderate or strong correlation for majority of these measurements. *r* = Pearson correlation coefficient and $R^2$ = regression analysis illustrate the correlation between manually and automatically calculated measurements.

### Comparing healthy and CRT patients strain patterns

**Figure 6:**
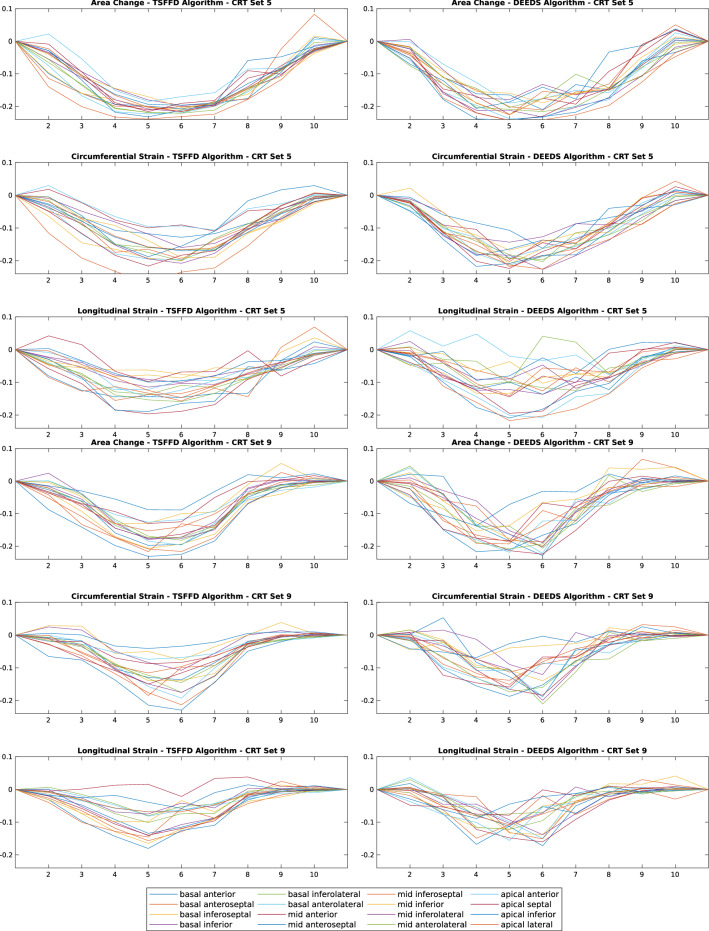
Example of three strains (area, circumferential, longitudinal) calculated from representative CRT datasets. Results from TSFFD and DEEDS are presented on the left and right columns, respectively. $\mathbf {X}$ axis displays the temporal frame in the cardiac cycle and $\mathbf {Y}$ axis is the calculated strain.

**Figure 7:**
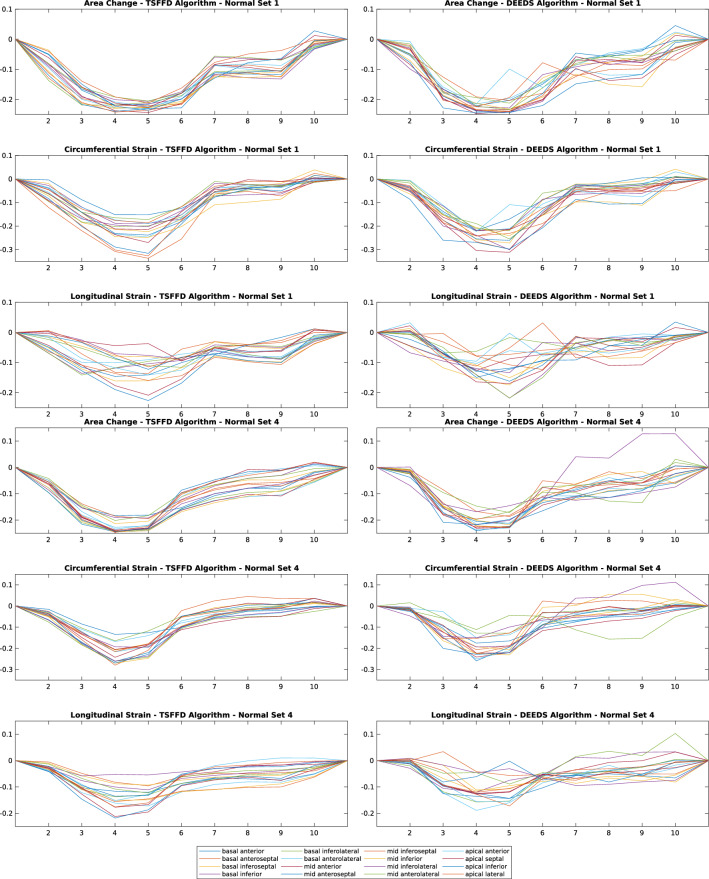
Example of three strains (area, circumferential, longitudinal) calculated from representative healthy datasets. Results from TSFFD and DEEDS are presented on the left and right columns, respectively. $\mathbf {X}$ axis displays the temporal frame in the cardiac cycle and $\mathbf {Y}$ axis is the calculated strain.

Previous MRI studies have found significant differences in the systolic dyssynchrony index (SDI) between healthy controls and CRT cases with dyssynchronous heart failure^26^. SDI is defined as the standard deviation of the time from cardiac cycle onset to minimum systolic volume in 16 LV segments^27^. We applied both of our registration algorithms to the healthy and the CRT cases to test their ability in detecting these differences. Figures 6 and 7 illustrate a subset of these results for CRT and healthy cases respectively. The plots visualise individual AHA curves computed using the two different registration tools, introduced previously. Strains computed by the TSFFD and DEEDS algorithms are pictured in the first and second columns respectively.

To determine whether the two sets of healthy and CRT data are significantly different from each other, we designed a two-sample *t*-test to compare the computed strains from each set. We therefore calculated three measurements from the SDI values to run the statistical analysis. The three measurements were: (1) time to the peak (T2P) of a curve, (2) time from the onset until a curve reaches 50% of its amplitude (TOS), and (3) magnitude of a curve (MAG) defined as the difference between its maximum and minimum peaks. TSFFD was more able to differentiate between normal and dyssynchronous contraction patterns, as magnitudes of curves for both circumferential and longitudinal strains revealed a significant difference with 0.0065 and 0.0386 *p*-values, respectively. TSFFD results also confirmed that the time from onset until area and circumferential curves reach 50% of their amplitudes were significantly different for healthy and CRT cases. Table 2 illustrates the results of all two-sample *t*-tests for strains computed using TSFFD and DEEDS methods.

**Table 2:** Two-sample *t*-test using strains computed from TSFFD and DEEDS.

Registration	TSFFD	DEEDS
Algorithm	16 Segments SDI	16 Segments SDI
Area change T2P	0.2372	0.3895
Area change TOS	**0.0096**	0.2470
Area change MAG	0.9629	0.2969
Circumferential T2P	0.0895	0.3926
Circumferential TOS	**0.0144**	0.0901
Circumferential MAG	**0.0065**	**0.0011**
Longitudinal T2P	0.6906	0.6190
Longitudinal TOS	0.3558	0.1993
Longitudinal MAG	**0.0386**	0.4550

### Effects of the number of reconstructed temporal frames

**Figure 8:**
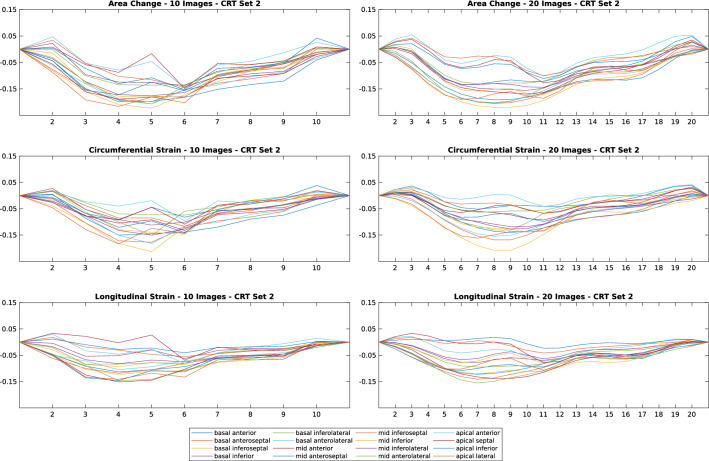
Effect of 20 frames over the cardiac cycle instead of 10 on the three strain (area, circumferential, longitudinal) curves. The plot is from one representative dataset. $\mathbf {X}$ axis displays the temporal frame in the cardiac cycle and $\mathbf {Y}$ axis is the calculated strain.

To keep processing times short, retrospective image reconstruction algorithms are usually used to generate $10-20$ temporal frames per cardiac cycle length^8,14,15^. We acquired 15 specific test datasets to evaluate the impact of number of frames on strain calculations from CCT. TSFFD with tuned hyperparameters were applied on the full dataset consisting of 20 images to obtain the transformation field. The same algorithm was then used to produce transformation fields based on half of the available images. Results showed that Time to the peak of curves (T2P) measurements varied between 5.05% and 5.57%, whereas magnitude of curves (MAG) had no significant difference. The most variation was observed for the time from the onset (TOS) measurements, with a maximum error of $8.51\pm 0.8$%. Figure 8 illustrates the effect of temporal frames on the strains.

## Discussion

In this study, we utilised retrospective gated CCT images for measuring regional cardiac mechanics using intensity-based registration techniques on clinical data. Our contributions include optimisation of two registration methods for tracking regional motion using manually annotated datasets, and deriving validated circumferential and longitudinal strains using large strain theory in addition to area, which has been the main measure examined in the literature^8,9,12,28^. The number of required temporal frames to calculate reliable strains was also examined. Our method does not require manual segmentation of the LV for every time frame in the cardiac cycle and completes the registration in approximately five minutes. All our workflow is available in an open-source platform to support the increased use of CCT datasets for analysing cardiac mechanics. Furthermore, we showed that the TSFFD method was more accurate than DEEDS in replicating differences in motion indices between healthy subjects and CRT patients. Tracking cardiac motion with TSFFD provided preliminary evidence that the lower frame rate of CCT does not demonstrably change the cardiac strain patterns. A higher number of reconstructed temporal frames resulted in smoother strain curves but did not change measurements such as magnitude of the curves.

### Radiation dose

Retrospective gated scanning of the entire cardiac cycle requires a significant amount of radiation, limiting wide application in studies and the standard clinical practice is to achieve prospective ECG-gated CCT for patients undergoing routine scanning in order to minimise the ionising radiation dose exposure. Despite this, there are certain indications where a full retrospective gated CCT scan is still required: for structural heart interventions such as transcatheter mitral valve planning, for patients with frequent ventricular ectopy or atrial fibrillation, for patients with suboptimal imaging from echocardiography, and/or for patients unable to undergo CMR scans due to implantable electronic devices, claustrophobia, and intolerance to the duration of the CMR scan owing to dyspnoea. We recognise that our proposed methodology should only be used, where full retrospective gated datasets are currently available. With the implementation of radiation dose reducing algorithms and modern scanners, we anticipate that radiation doses from this type of scans will further reduce, thereby our methodology may be employed for other patient populations at an acceptable ionising dose level.

We evaluated our registration techniques on patients with severe heart failure, who are known to have a significant difference in deformation from healthy controls^29^. Currently, these are one of the main patient groups in our institute receiving retrospective gated CCT due to the ionising radiation exposure. However, our hyperparameter optimised registration method can measure valvular plane motion to within $2.7\pm 0.99$ mm, estimated from the relevant automatically tracked anatomical landmarks in the validation datasets. This is expected to be sufficient to identify mild or moderate heart failure patients, who have been previously identified by a decrease in atrioventricular plane motion of up to 2–4 mm^30,31^.

### Effects of surface smoothing

We applied volume preserving smoothing operations on the temporal meshes in our workflow. This may have introduced error in tracking. We therefore performed an additional test and calculated the error between the manual segmentations and the constructed surface meshes. We observed that the mean error for all datasets was within the range of 0.5mm and 0.75mm. This error was very close to the voxel size of the images and was unlikely to have an impact on strain results.

### Hyperparameters optimisation

It became clear from our experiments that adopting default TSFFD hyperparameters^16^ for CMR were not optimal for CCT. They needed to be optimised to measure mechanics from CCT images. We discovered that the optimisation of TSFFD bending energy hyperparameter has an influential effect on the strain indices calculated. This is because too large a value would make the registration very stiff and too small a value would make it too sensitive to image artefacts. Analysis of measurements from anatomical landmarks enabled the selection of optimal value for the bending energy hyperparameter. Across all anatomical measurements, the error bounds for bending energy in the training sets were $[1.4\%, 11\%]$. The *Mitral Annulus Midpoint to head of the Anterolateral Papillary Muscle* measurement appeared to result in the largest error. Removing this measurement moves the upper bound on the training error to 8.22%.

It was further observed that the DEEDS algorithm could produce robust strain measures over a wider range of hyperparameter values. Across all anatomical measurements, the error bounds for the smoothness hyperparameter of registration in the training sets were $[3.13\%, 13.79\%]$. The *Mitral Annulus Midpoint to head of the Anterolateral Papillary Muscle* measurement had the largest error, as it was also the least reproducible annotation. Ignoring this measurement moves the upper bound on the training error to 11.45%.

CCT images are typically larger than CMR images. Therefore, choosing reasonable values for the resolution of control points in a multi-level free-form deformation setting has a significant effect on the speed of registration. We have reported the details of these resolutions in the registration hyperparameters section in the supplements.

### Validation error

Manual annotations can be used for validating medical image processing algorithms. However, the ability to consistently label anatomical features on repeating images has to be reliable. In our study, we required the manual annotation of the mitral valve midpoint. This is a point within the blood pool and has not obvious features. Identifying position of the midpoint in repeating images is thus difficult. Within our workflow, an automatic midpoint was calculated from the mitral valve landmarks placed on the annulus, whereas this point was labelled manually in OsiriX.

Measuring the *Mitral Annulus Midpoint to head of the Anterolateral Papillary Muscle* distance relies on the mitral valve landmarks. We found from the correlation results that there was a large and significant difference between the manual annotations and the automatically calculated values, suggesting that these measurements are not reliable. Based on the previous study by Banks et al.^24^, on average the distance from the mitral annulus midpoint to head of each papillary muscle (Anterolateral PM: $25.6\pm 4.5$mm, Posteromedial PM: $29.2\pm 3.9$mm) is smaller than the distance to the LV apex (Endocardial Apex: $90.9\pm 10.7$mm, Epicardial Apex: $96.4\pm 10.9$mm). This results in a larger effect of any error for the mitral annulus midpoint on the PM distances. Therefore, we need to consider the ease of labelling any tracked feature, as it was seen with the midpoint example, any error in placement can have an observable effect on the tracking accuracy. For the majority of the measurements (Figs. 4 and 5), the error in both of our registration algorithms was within or close to the bounds of the intra-observer error. This makes the tracking of the landmarks as accurate as possible within the observed measurement error.

TSFFD results from the validation data revealed that *Mitral Annulus Midpoint to Endocardial Border of the Left Ventricle (LV) Apex* demonstrates a good correlation with $r=0.86$ and $R^2=0.74$ along the longitudinal direction of the LV. Similarly, *Mitral Annulus Area* with $r=0.83$ and $R^2=0.69$ displays an acceptable correlation along the circumferential direction, reassuring the accuracy of calculated strains in these directions. DEEDS algorithm performed worse but still demonstrated a good correlation for *Mitral Annulus Midpoint to Endocardial Border of the Left Ventricle (LV) Apex* and reasonable results for *Mitral Annulus Area* measurements.

### Comparison to similar methods

Historically, computing regional cardiac mechanics from CCT have had limited applicability due to radiation and low temporal resolution. This has resulted in a substantial body of literature for echocardiography and CMR methods in comparison to CCT. For instance, Tee et al.^32^ utilised a simple method of block-matching technique originally developed for 2D ultrasound to estimate motion from CCT and to calculate circumferential and radial strains. Their study used a porcine infarct model and the correlation with harmonic phase (HARP^4^) in their work was moderate in the radial direction $(R^2=0.55)$ and weak in the circumferential direction $(R^2=0.4)$. 2D block-matching is unable to consider the through-plane motion and this can cause the method to be erroneous by not treating the volume as a whole. Our two proposed methods consider the full volume and describe strain in 3D space but are limited to quantifying regional function in the circumferential-longitudinal plane.

To verify the clinical usefulness of our methods, we compared our strain measurements in the six asymptomatic patients in the dataset with recordings of similar patients in the literature using MRI feature tracking, myocardial tagging, and speckle tracking echocardiography. Reported global end systolic circumferential strain varies between − 24.3% and −15% for MRI feature tracking and tagging techniques^33,34^, and between − 27.8% and − 20.9% for echocardiography^35^. This compares with our mean measurement of $-22.2\pm 2.4\%$. The reported ranges of longitudinal strain are between − 20.9% and − 15% for MRI feature tracking and tagging techniques^33,34^, and between − 22.1% and − 15.9% for echocardiography^35^. This compares with our mean measurement of $-15.8\pm 2.8\%$. Our CCT derived strain measurements are comparable with both MRI and echocardiography measurements in similar, but distinct, patient groups. Dedicated datasets with multi-modality imaging on the same patients would be required to confirm equivalence.

An example of measuring radial, circumferential, and principal strains from 4D CCT images in a canine infarct model includes the work of Wong et al.^36^ They used deformable image registration to track the LV endocardium and epicardium. They deformed a finite element model of the LV with the resulting displacements and proposed a method to detect the infarcted region using image intensity. The authors used a traditional B-spline transform registration implementation to wrap a source image into a target image. Like the DEEDS approach used in our work, there is no single way to apply these registration techniques to an image sequence without foundational modifications. The authors compared frame-to-frame and reference-frame formulations, and found similar accuracy in terms of infarct detection. However, they did not have any direct method of comparing tracking error. In our work, we can rely on our annotated datasets to measure the quality of our motion estimation.

Recently, Gupta et al.^13^ proposed Dice similarity coefficient and point-to-curve error to assess tracking accuracy of a deformable image registration technique for analysing LV motion from CCT datasets of 10 patients. However, their selected validation scores required the fully segmented left ventricle cavity from every phase in the cardiac cycle. Furthermore, they did not attempt to systematically examine the effects of optimising registration hyperparameters and their computation time of 35 min per patient for the entire cardiac cycle is higher than our average 5 min per patient using an Intel Xeon CPU with 32GB of memory. Lamash et al.^14^ work reported similar computation time of approximately 5 min to us on a standard CPU.

### Limitations

The observable dyssynchronous longitudinal plots in Fig. 7 for healthy subjects revealed that DEEDS struggles more than TSFFD in capturing longitudinal strains in datasets, where motion artefacts exist or low spatial resolution is unavoidable. DEEDS previously showed a high accuracy for registering anatomical structures in comparison to several state-of-the-art approaches^17^ but it was not originally designed for capturing a cyclic motion and therefore registering the cardiac motion using a frame-to-frame approach resulted in an accumulated error. We applied all registrations to the original reference frame to alleviate this error. However, this increases the differences between target and reference frames, potentially making the registration problem more challenging. DEEDS does not include the continuous time constraint introduced in the TSFFD algorithm for 4D registration and hence computes noisier strain indices.

Our calculations were limited to area, circumferential and longitudinal strains. We did not consider radial strain, as segmenting myocardium can be problematic due to image artefacts from existing leads in the CRT datasets. Moreover, segmenting and tracking the feature rich endocardium from CCT datasets is less error prone from adequate number of frames and has also been previously proposed as a good indicator of optimal CRT pacing lead location based on acute hemodynamic response^15^. Nonetheless, it is worth to mention that our registration methods generate motion displacement fields for the entire image space and therefore, if reliable segmentation of myocardium is obtained, the rest of pipeline can be used to calculate radial strain without any major processing step.

The number of available CCT datasets are limited due to the problem of radiation dose. Large annotated sets are also difficult to acquire. Although our dataset of 24 patients is larger than most of relevant work in the literature^12,13^, we acknowledge a bigger patient cohort will be needed in future to generalise our findings.

## Supplementary Information

Supplementary Information.

## References

[1] Tee M, Noble JA, Bluemke DA (2013). Imaging techniques for cardiac strain and deformation: comparison of echocardiography, cardiac magnetic resonance and cardiac computed tomography. Exp. Rev. Cardiovasc. Ther..

[2] Buss S (2014). Quantitative analysis of left ventricular strain using cardiac computed tomography. Eur. J. Radiol..

[3] Aletras AH, Ding S, Balaban RS, Wen H (1999). DENSE: Displacement encoding with stimulated echoes in cardiac functional MRI. J. Magnet. Resonan..

[4] Osman NF, Kerwin WS, McVeigh ER, Prince JL (1999). Cardiac motion tracking using cine harmonic phase (HARP) magnetic resonance imaging. Magnet. Resonan. Med..

[5] Scatteia A, Baritussio A, Bucciarelli-Ducci C (2017). Strain imaging using cardiac magnetic resonance. Heart Failure Rev..

[6] Mak GS, Truong QA (2012). Cardiac CT: Imaging of and through cardiac devices. Curr. Cardiovasc. Imaging Rep..

[7] Daubert JC (2012). 2012 EHRA/HRS expert consensus statement on cardiac resynchronization therapy in heart failure: implant and follow-up recommendations and management. Europace.

[8] Pourmorteza A, Schuleri K. H, Herzka D. A, Lardo A. C, McVeigh E. R (2012). A new method for cardiac computed tomography regional function assessment: stretch quantifier for endocardial engraved zones (SQUEEZ). Circ. Cardiovasc. Imaging.

[9] Pourmorteza A, Chen MY, van der Pals J, Arai AE, McVeigh ER (2016). Correlation of CT-based regional cardiac function (SQUEEZ) with myocardial strain calculated from tagged MRI: an experimental study. Int. J. Cardiovasc. Imaging.

[10] Myronenko A, Song X (2010). Point set registration: Coherent point drift. IEEE Trans. Pattern Anal. Mach. Intell..

[11] Stebbing, R. *Model-based segmentation methods for analysis of 2D and 3D ultrasound images and sequences*. Ph.D. thesis (University of Oxford, 2014).

[12] Vigneault DM, Pourmorteza A, Thomas ML, Bluemke DA, Noble JA (2018). SiSSR: Simultaneous subdivision surface registration for the quantification of cardiac function from computed tomography in canines. Med. Image Anal..

[13] Gupta V (2018). Automated three-dimensional tracking of the left ventricular myocardium in time-resolved and dose-modulated cardiac ct images using deformable image registration. J. Cardiovasc. Comput. Tomogr..

[14] Lamash Y, Fischer A, Carasso S, Lessick J (2015). Strain analysis from 4-d cardiac ct image data. IEEE Trans. Biomed. Eng..

[15] Behar JM (2017). Comprehensive use of cardiac computed tomography to guide left ventricular lead placement in cardiac resynchronization therapy. Heart Rhythm.

[16] Shi W (2013). Temporal sparse free-form deformations. Med. Image Anal..

[17] Heinrich MP, Jenkinson M, Brady M, Schnabel JA (2013). MRF-based deformable registration and ventilation estimation of lung CT. IEEE Trans. Med. Imaging.

[18] Rueckert D (1999). Nonrigid registration using free-form deformations: Application to breast MR images. IEEE Trans. Med. Imaging.

[19] Schnabel, J. A. *et al.* A generic framework for non-rigid registration based on non-uniform multi-level free-form deformations. In *Medical Image Computing and Computer Assisted Intervention (MICCAI)*, Vol. 2208, 573–581. Lecture Notes in Computer Science (2001).

[20] Cerqueira MD (2002). Standardized myocardial segmentation and nomenclature for tomographic imaging of the heart. Circulation.

[21] Modat M (2010). Fast free-form deformation using graphics processing units. Comput. Methods Programs Biomed..

[22] Klein S, Staring M, Murphy K, Viergever MA, Pluim JP (2010). Elastix: a toolbox for intensity-based medical image registration. IEEE Transactions on Medical Imaging.

[23] Heinrich, M. P., Jenkinson, M., Papiez, B. W., Brady, S. M. & Schnabel, J. A. Towards realtime multimodal fusion for image-guided interventions using self-similarities. in *Medical Image Computing and Computer Assisted Intervention (MICCAI)*, Vol. 8149, 187–194 *Lecture Notes in Computer Science* (Springer, 2013). 10.1007/978-3-642-40811-3_24 24505665

[24] Banks, T. *et al.* Automated quantification of mitral valve geometry on multi-slice computed tomography in patients with dilated cardiomyopathy—Implications for transcatheter mitral valve replacement. *J. Cardiovasc. Comput. Tomogr.* **(in press)** (2018). 10.1016/j.jcct.2018.04.003 29747948

[25] Rosset A, Spadola L, Ratib O (2004). OsiriX: An open-source software for navigating in multidimensional DICOM images. J. Digit. Imaging.

[26] Sohal M (2014). A prospective evaluation of cardiovascular magnetic resonance measures of dyssynchrony in the prediction of response to cardiac resynchronization therapy. J. Cardiovasc Magnet. Resonan..

[27] Kapetanakis S (2005). Real-time three-dimensional echocardiography. Circulation.

[28] McVeigh, E. R. *et al.* Regional myocardial strain measurements from 4DCT in patients with normal LV function. *J. Cardiovasc. Comput. Tomogr.* **(in press)** (2018). 10.1016/j.jcct.2018.05.002 PMC7458583 29784623

[29] Cheung Y (2012). The role of 3d wall motion tracking in heart failure. Nat. Rev. Cardiol..

[30] Willenheimer R, Cline C, Erhardt L, Israelsson B (1997). Left ventricular atrioventricular plane displacement: an echocardiographic technique for rapid assessment of prognosis in heart failure. Heart.

[31] Rangarajan, V. *et al.* Left ventricular long axis function assessed during cine-cardiovascular magnetic resonance is an independent predictor of adverse cardiac events. *J. Cardiovasc. Magnet. Resonan.* **18** (2016). 10.1186/s12968-016-0257-y PMC4897936 27266262

[32] Tee MW (2015). Regional strain analysis with multidetector CT in a swine cardiomyopathy model: Relationship to cardiac MR tagging and myocardial fibrosis. Radiology.

[33] Vo H, Marwick T, Negishi K (2018). MRI-Derived myocardial strain measures innormal subjects. JACC: Cardiovasc. Imaging.

[34] Jeung M (2012). Myocardial tagging with MR imaging: Overview of normal and pathologic findings. RadioGraphics.

[35] Yingchoncharoen T, Agarwal S, Popovic Z, Marwick T (2013). Normal ranges of left ventricular strain: A meta-analysis. J. Am. Soc. Echocardiogr..

[36] Wong KC (2016). Regional infarction identification from cardiac CT images: A computer-aided biomechanical approach. Int. J. Comput. Assist. Radiol. Surg..

