# Supplementary material for: Hyperparameter optimisation and validation of registration algorithms for measuring regional ventricular deformation using retrospective gated computed tomography images
Source: Sci Rep. 2021 Mar 11;11:5718. doi: 10.1038/s41598-021-84935-x (PMC7952400; doi:10.1038/s41598-021-84935-x)
Supplement: Supplementary file 1 — Supplementary Information. [file 41598_2021_84935_MOESM1_ESM.pdf]

Hyperparameter Optimisation and Validation of Registration Algorithms for Measuring Regional Ventricular Deformation using Retrospective Gated Computed Tomography Images

Orod Razeghi[1,*], Mattias Heinrich[2], Thomas E Fastl[1], Cesare Corrado[1], Rashed Karim[1], Adelaide De Vecchi[1], Tom Banks[3], Patrick Donnelly[4], Jonathan M Behar[1,5], Justin Gould[1,3], Ronak Rajani[1,3], Christopher A Rinaldi[1,3], and Steven Niederer[1]

[1]School of Biomedical Engineering and Imaging Sciences, King's College London, London, United Kingdom
[2]Insitute of Medical Informatics, University of Lübeck, Lübeck, Germany
[3]Department of Cardiology, Guy's and St Thomas' NHS Foundation Trust, London, United Kingdom
[4]South Eastern Health and Social Care Trust
[5]Department of Electrophysiology, Barts Heart Centre, London, United Kingdom
[*]orod.razeghi@kcl.ac.uk

Supplementary Information

Calculation of Strain

Deformation in mechanics is the transformation of a geometry from a reference configuration Ω_0 to a current configuration Ω. We describe the geometry using a Delaunay triangulation and the displacement with linear Lagrange interpolation. In that follows, we denote the coordinates in the reference configuration with $\mathbf{X} \in \mathbb{R}^3$, the coordinates in the current configuration with $\mathbf{x} \in \mathbb{R}^3$, and with $\mathbf{F}$ the Jacobian that locally maps the reference configuration into the current configuration. As we describe both geometries with a Delaunay triangulation and the displacement with linear Lagrange interpolation, we denote the coordinates of the unitary simplex with $\boldsymbol{\xi} \in \mathbb{R}^2$. Quantities are illustrated in Figure A1.

Transformations between the unitary simplex and the current or the reference configurations define the covariant basis, characterising the local tangent spaces. Covariant basis of the current and the reference configurations mutually transform through the deformation gradient tensor:

$$\frac{\partial \boldsymbol{x}}{\partial \xi_I} = \frac{\partial \boldsymbol{x}}{\partial X_k}\frac{\partial X_k}{\partial \xi_I}, \qquad I = 1, 2. \tag{1}$$

The local normal to both surfaces are defined as:

$$\mathbf{n_x} = \frac{\frac{\partial \mathbf{x}}{\partial \xi_1} \times \frac{\partial \mathbf{x}}{\partial \xi_2}}{\| \frac{\partial \mathbf{x}}{\partial \xi_1} \times \frac{\partial \mathbf{x}}{\partial \xi_2} \|} \quad \text{and} \quad \mathbf{n_X} = \frac{\frac{\partial \mathbf{X}}{\partial \xi_1} \times \frac{\partial \mathbf{X}}{\partial \xi_2}}{\| \frac{\partial \mathbf{X}}{\partial \xi_1} \times \frac{\partial \mathbf{X}}{\partial \xi_2} \|}. \tag{2}$$

The local space $\mathbf{J}$ in the reference configuration is then mapped to the local space $\mathbf{j}$ in the current configuration with the Jacobian, as follows:

$$\underbrace{\begin{bmatrix} \frac{\partial x_1}{\partial \xi_1} & \frac{\partial x_1}{\partial \xi_2} & n_1^x \\ \frac{\partial x_2}{\partial \xi_1} & \frac{\partial x_2}{\partial \xi_2} & n_2^x \\ \frac{\partial x_3}{\partial \xi_1} & \frac{\partial x_3}{\partial \xi_2} & n_3^x \end{bmatrix}}_{\mathbf{j}} = \underbrace{\begin{bmatrix} \frac{\partial x_1}{\partial X_1} & \frac{\partial x_1}{\partial X_2} & \frac{\partial x_1}{\partial X_3} \\ \frac{\partial x_2}{\partial X_1} & \frac{\partial x_2}{\partial X_2} & \frac{\partial x_2}{\partial X_3} \\ \frac{\partial x_3}{\partial X_1} & \frac{\partial x_3}{\partial X_2} & \frac{\partial x_3}{\partial X_3} \end{bmatrix}}_{\mathbf{F}} \underbrace{\begin{bmatrix} \frac{\partial X_1}{\partial \xi_1} & \frac{\partial X_1}{\partial \xi_2} & n_1^X \\ \frac{\partial X_2}{\partial \xi_1} & \frac{\partial X_2}{\partial \xi_2} & n_2^X \\ \frac{\partial X_3}{\partial \xi_1} & \frac{\partial X_3}{\partial \xi_2} & n_3^X \end{bmatrix}}_{\mathbf{J}}. \tag{3}$$

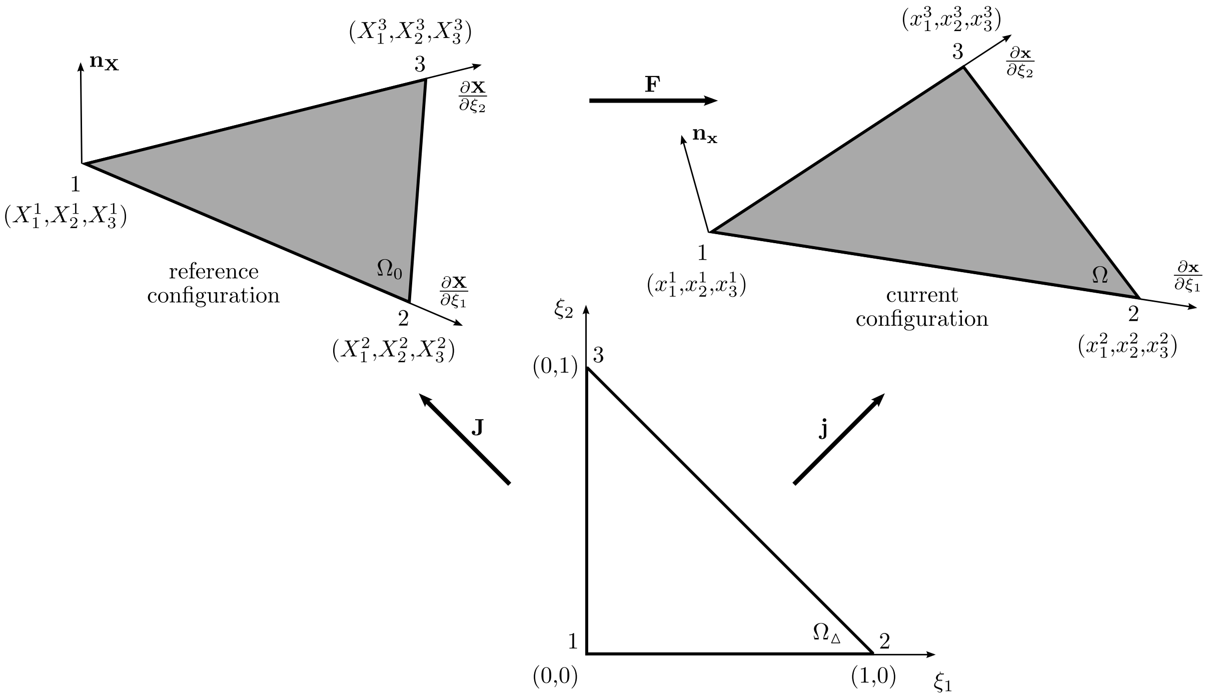

Figure A1. Isoparametric mapping of the deformation between the simplex $\Omega_\triangle$, the reference configuration Ω_0, and the current configuration Ω induced by the deformation.

Rearrangement of this relation to express the deformation gradient leads to: $\mathbf{F} = \mathbf{j}\mathbf{J}^{-1}$. Finally, the Green-Lagrange strain tensor $\mathbf{E}$ is calculated by equation:

$$\mathbf{E} = \frac{1}{2}(\mathbf{F}^{\mathrm{T}}\mathbf{F} - \mathbf{I}), \tag{4}$$

where $\mathbf{I}$ is the identity tensor. Both tensors quantities, $\mathbf{F}$ and $\mathbf{E}$, are constant throughout the 3-node triangular element because of the linear shape functions.

Strain Tests

Computation of the Green-Lagrange strain tensor $\mathbf{E}$ is necessary for calculation of circumferential and longitudinal strains. We verified the correctness of the implementation using idealised problems. The triangular meshes deployed in these tests were generated to represent the respective geometry in the reference configuration and artificial finite deformations were applied to the mesh vertices to obtain the current configuration. The results provided for verification include the deformation gradient tensor $\mathbf{F}$ and the Green-Lagrange strain tensor $\mathbf{E}$ in the global and the element coordinate system, respectively. Both measures are provided for the last element in each problem.

Tensile Test

A square (3 x 3 cm) was meshed using triangular elements of approximately 1 mm edge length resulting in 1235 vertices and 2348 elements. A biaxial deformation field (see Figure A2(a)) was applied to verify the mathematical description derived in the method's section by:

$$x_1 = 1.300 \cdot X_1,$$
$$x_2 = 1.100 \cdot X_2,$$
$$x_3 = X_3,$$

where x_i and X_i, with $i = 1, 2, 3$, are the coordinates in the current and reference configuration, respectively. The implementation of the Green-Lagrange strain tensor provided:

$$\mathbf{F} = \begin{bmatrix} 1.300 & 0.000 & 0.000 \\ 0.000 & 1.100 & 0.000 \\ 0.000 & 0.000 & 1.000 \end{bmatrix}, \qquad \mathbf{E_e} = \begin{bmatrix} 0.000 & 0.000 & 0.000 \\ 0.000 & 0.345 & 0.000 \\ 0.000 & 0.000 & 0.105 \end{bmatrix}.$$

where $\mathbf{F}$ is in the global Cartesian coordinate system and $\mathbf{E_e}$ is in the cylindrical element coordinate system with $\mathbf{v}_{\text{ab}} = [0\ 1\ 0]^{\text{T}}$. The results suggest the correct implementation under conditions where the element's normal remained fixed and strains were aligned with the rectangular Cartesian coordinates.

Shear Test

A square (3 x 3 cm) was meshed using triangular elements of approximately 1 mm edge length resulting in 1235 vertices and 2348 elements. A simple shear deformation field (see Figure A2(b)) was applied to verify the mathematical description derived in the method's section by:

$$x_1 = X_1 + 0.333 \cdot X_2,$$
$$x_2 = X_2,$$
$$x_3 = X_3,$$

where x_i and X_i, with $i = 1, 2, 3$, are the coordinates in the current and reference configuration, respectively. The implementation of the Green-Lagrange strain tensor provided:

$$\mathbf{F} = \begin{bmatrix} 1.000 & 0.333 & 0.000 \\ 0.000 & 1.000 & 0.000 \\ 0.000 & 0.000 & 1.000 \end{bmatrix}, \qquad \mathbf{E_e} = \begin{bmatrix} 0.000 & 0.000 & 0.000 \\ 0.000 & 0.000 & 0.167 \\ 0.000 & 0.167 & 0.056 \end{bmatrix}.$$

where $\mathbf{F}$ is in the global Cartesian coordinate system and $\mathbf{E_e}$ is in the cylindrical element coordinate system with $\mathbf{v}_{\text{ab}} = [0\ 1\ 0]^{\text{T}}$. The results suggest the correct implementation under conditions where shear strains were aligned with the rectangular Cartesian coordinates.

Tube Test

A tube (3 x 3 cm) was meshed using triangular elements of approximately 1 mm edge length resulting in 3416 vertices and 6640 elements. A multi dimensional deformation field (see Figure A2(c)) was applied to verify the mathematical description derived in the method's section by:

$$x_1 = 1.200 \cdot X_1,$$
$$x_2 = 1.200 \cdot X_2,$$
$$x_3 = 1.100 \cdot X_3,$$

where x_i and X_i, with $i = 1, 2, 3$, are the coordinates in the current and reference configuration, respectively. The implementation of the Green-Lagrange strain tensor provided:

$$\mathbf{F} = \begin{bmatrix} 1.000 & 0.007 & 0.002 \\ 0.007 & 1.200 & 0.000 \\ 0.000 & 0.000 & 1.100 \end{bmatrix}, \qquad \mathbf{E_e} = \begin{bmatrix} 0.000 & 0.000 & 0.000 \\ 0.000 & 0.220 & 0.000 \\ 0.000 & 0.000 & 0.105 \end{bmatrix}.$$

where $\mathbf{F}$ is in the global Cartesian coordinate system and $\mathbf{E_e}$ is in the cylindrical element coordinate system with $\mathbf{v}_{\text{ab}} = [0\ 0\ 1]^{\text{T}}$. The results suggest the correct implementation under conditions where strains on surfaces are not aligned with the Cartesian coordinate system.

As an additional test, the surface of the same tube was labelled with standard AHA segments and an identical multi dimensional deformation field was applied to the 5th and 6th time frames in the sequence to verify the mathematical derivation. It is clear from Figure A3 that all the AHA curves corresponding to different regions of the tube expand simultaneously and illustrate a correct value of strain at the given time point in the sequence.

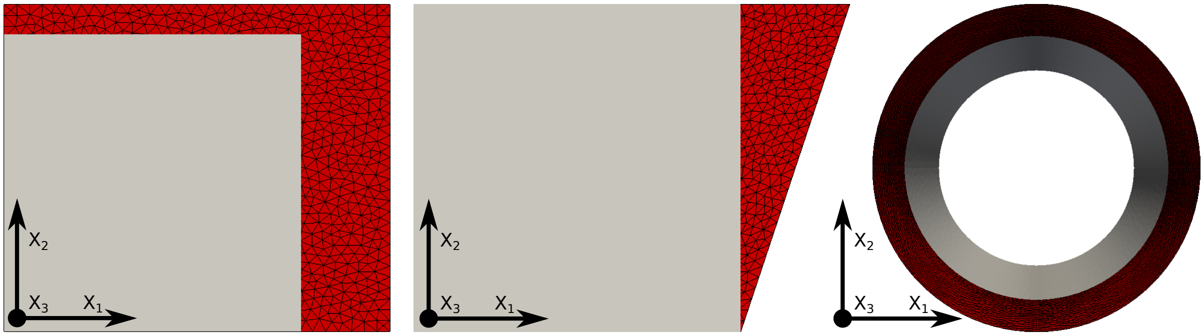

(a) Tensile Test (b) Shear Test (c) Tube Test

Figure A2. Reference (grey) and current (red) configuration of all described test problems implemented for verification purposes. The triangular mesh is indicated in the current configuration. The coordinate origin of all geometries is the lower left corner, except for the tube tests, which is located at the centre.

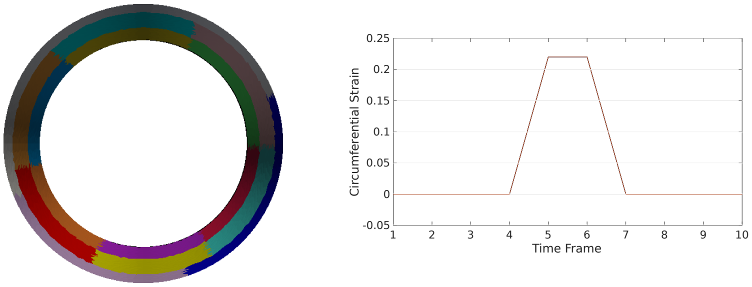

Figure A3. Simultaneous contraction of AHA curves in the tube test problem implemented for verification purposes.

Left Ventricular Mesh Test

Previous verifications provide confidence of the correct derivation and implementation of the Green-Lagrange strain tensor **E** for triangular elements. The following additional test was implemented to provide special cases of the presented problem. Endocardium of an LV was meshed using 32952 vertices and 65900 triangular elements. A multi dimensional deformation field was applied to all the 10 input meshes in the sequence to verify the mathematical description derived in the previous section by:

$$
\begin{aligned}
x_1 &= 0.800 \cdot X_1, \\
x_2 &= 0.800 \cdot X_2, \\
x_3 &= 0.800 \cdot X_3,
\end{aligned}
$$

where x_i and X_i, with $i = 1,2,3$, are the coordinates in the middle time frames configurations of the sequence and the reference configuration, respectively. A monotonic increase and decrease of these values was also applied to the rest of the configurations corresponding to all time points in the sequence. The implementation of the Green-Lagrange strain tensor E provided a monotonic contraction of all AHA segments in the LV suggesting a correct implementation (see Figure A4).

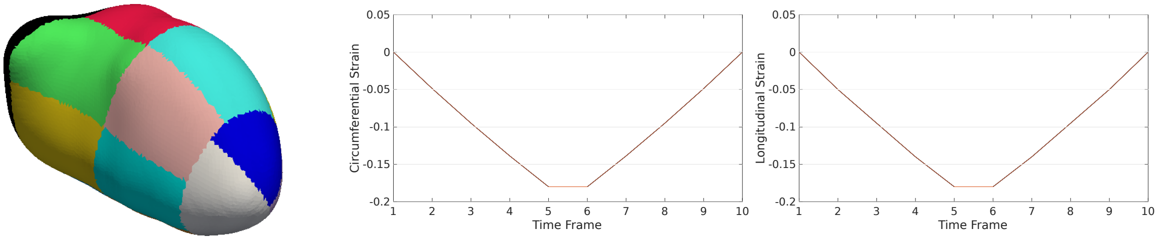

Figure A4. Monotonic contraction of AHA curves in the left ventricular mesh test problem implemented for verification purposes.

Registration Hyperparameters Optimisation

Figure A5 illustrates the results of an exhaustive grid search for the optimal values of bending energy and sparsity weight in TSFFD registration framework. Dual optimisation of sparsity weight does not converge to a local minima. Similarly, Figure A6 demonstrates the effect of optimising Θ from DEEDS on 10 training sets. This hyperparameter can be increased to obtain smoother transforms and decreased to make the registration more aggressive. The search in the hyperparameter's space does not reveal a significant trend.

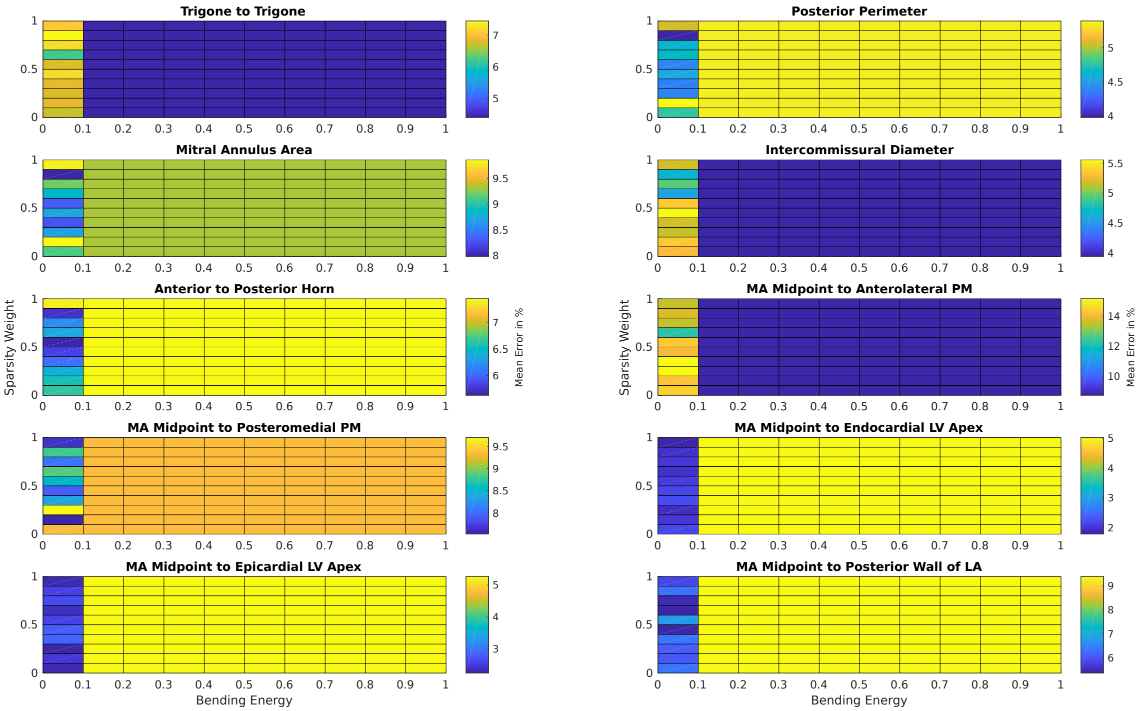

Figure A5. Dual optimisation of registration hyperparameters and their effect on endocardial tracking accuracy. The accuracy was measured at selected anatomical sites. The **X** and **Y** axes in the figure correspond to bending energy and sparsity weight, respectively. The colour code represents the average error in percentage on the training sets. Bending energy with small values has the most observable effect on the accuracy. Sparsity weight does not reveal a trend.

Registration Hyperparameters

Below is the full list of default hyperparameters for both TSFFD and DEEDS registration methods.

```
TSFFD
[ input ] Padding value = −1024
[ optimisation ] Energy preconditioning = 0.001, Divide data terms by initial value = No
[ optimisation ] Interpolation mode = Linear, Epsilon = −1e−6
[ Level 1 ] Blurring [mm] = 03, Resolution [mm] = 05 05 05, Min length of steps = 0.01, Max length of steps = 4
[ Level 2 ] Blurring [mm] = 05, Resolution [mm] = 10 10 10, Min length of steps = 0.01, Max length of steps = 4
[ Level 3 ] Blurring [mm] = 10, Resolution [mm] = 20 20 20, Min length of steps = 0.01, Max length of steps = 4
[ Level 4 ] Blurring [mm] = 20, Resolution [mm] = 40 40 40, Min length of steps = 0.01, Max length of steps = 8
```

```
DEEDS
–G grid spacing for each level = 8x7x6x5x4
–L maximum search radius = 8x7x6x5x4
–Q quantisation step = 5x4x3x2x1
```

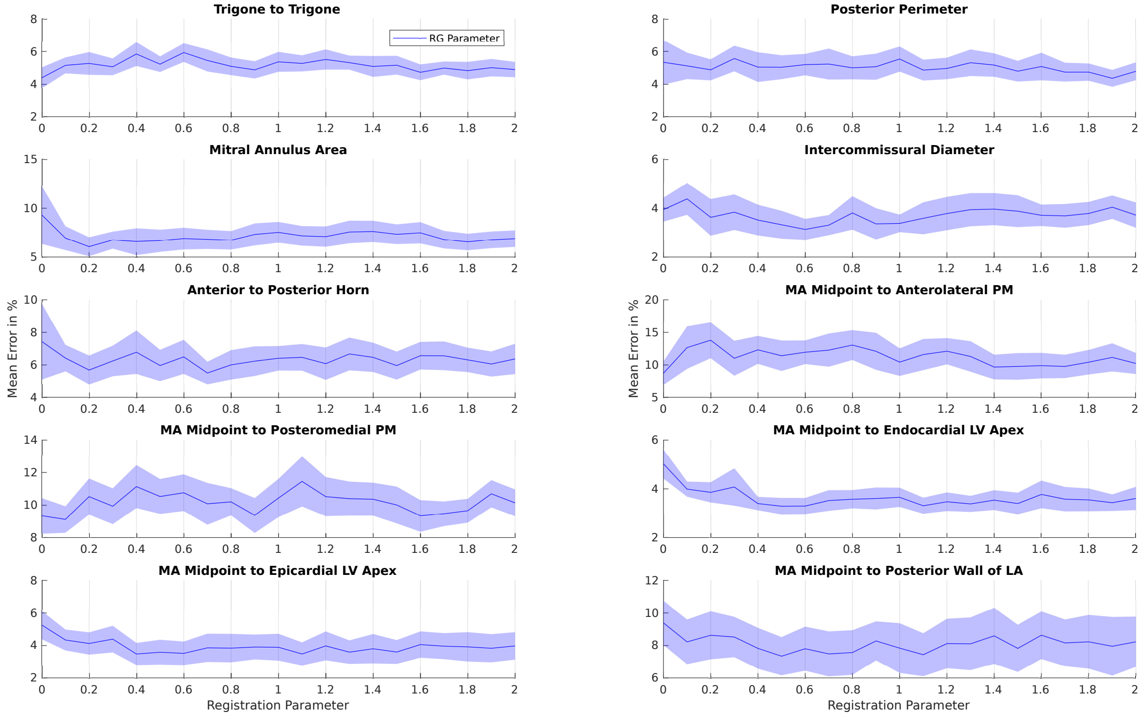

Figure A6. Optimisation of the registration hyperparameter Θ on the training sets and its effect on endocardial tracking accuracy. The accuracy was measured at selected anatomical sites. **X** axis is the examined range for the registration hyperparameter, whereas **Y** axis displays the average error in percentage. Standard error is illustrated as a shaded region.
